# Cost of “Ideal Minimum Integrated Care” for Type 2 Diabetes and Hypertension Patients in Cambodia Context: Provider Perspective

**DOI:** 10.5334/ijic.7682

**Published:** 2024-11-08

**Authors:** Sokunthea Yem, Srean Chhim, Edwin Wouters, Josefien Van Olmen, Por Ir, Grace Marie Ku

**Affiliations:** 1 National Institute of Public Health (NIPH), Phnom Penh, Cambodia; 2 Centre for Population, Family & Health, Department of Social Sciences, University of Antwerp, Antwerp, Belgium; 3 Centre for Health Systems Research & Development, University of the Free State, Bloemfontein, South Africa; 4 Department of Family Medicine and Population Health (FAMPOP), Faculty of Medicine and Health Sciences, University of Antwerp, Belgium; 5 Department of Public Health, Institute of Tropical Medicine, Antwerp, Belgium; 6 Department of Gerontology, Faculty of Medicine and Pharmacy, Vrije Universiteit Brussel, Belgium

**Keywords:** ideal minimum integrated care, scaling-up, cost modelling, built-model, input parameters

## Abstract

**Introduction:**

As in other countries worldwide, Diabetes mellitus type 2 (T2D) and hypertension (HTN) prevalence is increasing in Cambodia. The country is examining models to scale-up integrated T2D and HTN care. However, costs of integrated care in this setting are not yet well-understood. Thus, we modelled the cost of an “Ideal Minimum Integrated Care” (IMIC) package (detection, diagnosis, treatment + health education, self-management and follow-up) for T2D and HTN in Cambodia.

**Description:**

We visualised a package – IMIC – of effective interventions for T2D and HTN inspired by SCUBY-ICP and PEN. WHO NCD and HEART Costing Tools were adapted to estimate annual total IMIC intervention cost per health centre, cost per case and cost per capita.

**Discussion:**

Cost of the IMIC provides information on costs to aid decision-making on implementation. The Excel-based costing tool is easy to accomplish and can be replicated to provide more accurate results by using more precise actual input data, once these are available in the country.

**Conclusion:**

The projected costs of IMIC for T2D and HTN in Cambodia provides evidence to informed decision-making of relevant actors in implementing scale-up of IMIC for T2D and HTN. The model can be used in countries with similar context to calculate costs of integrated care.

## Introduction

Diabetes mellitus type 2 (T2D) and hypertension (HTN) are two of the most prevalent chronic diseases. They often co-occur and are both considered risk factors for cardiovascular diseases (CVDs), the leading cause of death worldwide [1]. Globally, the prevalence of T2D and HTN are 9.3% and 32% respectively [2,3]. About 79% of people with T2D and about 66% of people with HTN are found in low- and middle-income countries (LMICs) [4,5]. The prevalence of these two conditions increases at a faster rate in LMICs due to social, demographic, cultural and nutritional transitions including aging populations, urbanization, dietary changes, physical inactivity, increasing trends in overweight and obesity, and inadequacies of healthcare systems. In Cambodia, the prevalence of diabetes and hypertension among adults aged 40-years or older in 2020 was 12% and 27% respectively [6,7]. In addition to the disease burden, T2D and HTN pose substantial global and country economic burdens [8,9]. About 30% (US$84 million) of the national health budget was spent on NCDs in Cambodia in 2018 [10]. CVD, with hypertension as the top risk factor [11], accounted for 13.4% (US$38 million) and diabetes accounted for 4% (US$11 million). Furthermore, T2D and HT also posed indirect costs amounting to economic burden of around US$171 million [10].

A set of cost-effective evidence-based interventions known as the integrated care package (ICP)—detection and diagnosis, treatment, health education, self-management, and collaboration between caregivers—has been introduced, modified, and put into practice in many countries to address the burdens of T2D and HTN [12,13]. In Cambodia, the National Strategic Plan for the Prevention and Control of Noncommunicable Diseases 2013–2020 was established to promote integration of the management of NCDs by demonstrating and implementing the Package of Essential NCD Interventions in Primary Care (PEN). The ICP and PEN have commonalities; however, PEN has additional activities, eg. CVD risk screening and stratification. The viability and cost-effectiveness of PEN have been evaluated in several low-resource settings. However, the package’s implementation capacity, cost of implementation, and effectiveness are still doubtful [14]. Some challenges to the implementation of the PEN standard operating procedures for T2D and HTN in Cambodia have been demonstrated. These include low service coverage, insufficient human resource capacity, inadequate medicine supply, problematic documentation of patients’ data, and lack of care continuity [15,16]. PEN implementation varies between health centres (HC); some only do passive screening while others actively screen, a few initiate treatments while considerable numbers refer patients to hospitals for management. Additionally, the PEN is an ongoing, iterative care delivery model that is constantly modified. While it is possible to estimate the costs of actual implementation within a certain point in time, this would not reflect the cost of the actual and full PEN package. Thus, we propose a built-model for cost-estimation: the Ideal Minimum Integrated Care (IMIC).

Adapted from the SCUBY ICP [12] and the PEN, the IMIC is a package composed of effective interventions for T2D and HTN, namely screening including CVD risk stratification, healthy lifestyle counselling, treatment, self-management education and support (SMES), regular follow-up and community-based/home visits, that would contribute to better care of T2D and HTN in Cambodia [17,18,19,20,21].

The country is exploring ways to scale-up T2D and HTN care; it is highly important to take into consideration financing to support implementation. There are competing priorities in the investment of the national health budget and knowing the costs of different healthcare services is crucial for evidence-informed decision-making, for efficient and fair allocation of resources [22]. However, the supply side cost of interventions in Cambodia is not well-informed [23]. Therefore, this costing study provides, to the extent possible, a comprehensive estimate of the costs of an ideal minimum integrated care package for T2D, HTN and CVD risks, from the provider perspective.

## Ethics Approval

This study is a component of the SCUBY project, the protocol of which was approved by the Cambodian National Ethics Committee for Health Research (ref: 115). All necessary permissions and authorizations for the involvement of the MoH and participating HCs were secured.

## Description of Service delivery model: The Ideal Minimum Integrated Care

**Figure 1:**
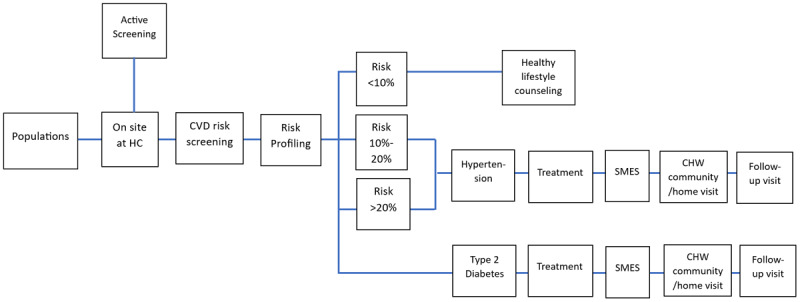
Activity components of “Ideal Minimum Integrated Care” for T2D and HTN. SMES – self-management education and support.

We conceptualized an IMIC patient flow (Figure 1), following healthcare provider activities: active screening, CVD risk screening and profiling, lifestyle counselling (for those who test negative for diabetes or have <10% CVD risk), treatment, SMES, regular follow-up and community-based/home visit. According to the flow, everyone arriving at the HC goes through triage for registration. Medical history-taking and clinical examination are performed by medical doctor/nurse. After the risk score is calculated and condition(s) are identified, HTN and/or T2D are managed according to country standards and guidelines (**Box 1**) [24,25].

Box 1. T2D and HTN national standard operating procedure and T2D clinical guideline in Cambodia*

*National standard operating procedure for T2D and HTN management in primary care 2019:*

Adults are defined to have hypertension if systolic blood pressure (SBP) is 140mmHg or over in repeated measurements. Drug treatment may be needed; amlodipine 5mg and hydrochlorothiazide (starting 12.5 mg, increasing to 25 mg per day) can be started at the HC. HTN Patients with a CVD risk between 10%–20% or greater and T2D patients require follow-up visit. During follow-up visit, laboratory testing, healthy lifestyle counselling and SMES are provided.

*Clinical Guideline for T2D*

Adults are defined to have type 2 diabetes if fasting blood glucose values are over 126mg/dl or random blood glucose values are over 200mg/dl. Lifestyle modification and/or drug treatment will be initiated by starting with low dose of metformin and/or glibenclamide and increase gradually every few weeks (metformin dose 500mg-2000mg in 2–3 divided doses per day after meals and glibenclamide dose 2.5–20mg 1–2 time with meal).

*(1) National standard operating procedure for diabetes and hypertension [24], (2) Clinical practice guidelines for type 2 diabetes [25]

Activities in the community such as active screening and community/home visits are performed by CHWs and are included in this costing. Other health problems or conditions that a health centre cannot clinically manage are referred to higher levels of care, e.g. referral or provincial hospital and were not costed in this model.

## Method

This is a case study utilising cost modelling to determine the supply-side (provider) cost of IMIC implementation in Cambodia.

### Data sources

This study retrospectively collected input data relevant to the IMIC components. Routine data of 2019 was collected from nine HCs in three different provinces; we also conducted key informant interviews and observation to collect data on turnaround times. The facilities were purposively selected based on PEN implementation and the availability of the input data. Table 1 shows relevant characteristics of the 9 study health facilities.

**Table 1:** Characteristics of study sites based on T2D and HTN activities.


HEALTH FACILITY	T2D			HTN			NO. OF STAFF TRAINED AND DELIVERING CARE FOR T2D AND HTN				
		
	NO. OF PEOPLE SCREENED	NO. OF PEOPLE WITH T2D CARED FOR	NO. OF VISITS / CONTACTS	NO. OF PEOPLE SCREENED	NO. OF PEOPLE WITH HTN CARED FOR	NO. OF VISITS / CONTACTS	NURSE	MW	MD	OTHER	TOTAL

Health center (A)*											

Prey Pnov HC	50	?	?	50	170	850	4	0	0	0	4

Prey Sneat HC (HSD)	125	?	?	0	143	715	2	0	0	1	3

Samroang (HSD)	144	?	?	0	90	450	2	1	0	0	3

Leay Bo HC	81	?	?	81	72	216	3	0	0	0	3

Health center (B)**											

Kvav HC	108	?	-	108	49	147	3	0	0	0	3

Reab HC	850	?	300	0	145	722	2	2	0	1	5

Kagnchom HC	403	13	23	403	122	434	4	3	0	0	7

Kompong Kdey HC	?	31	110	?	93	462	2	1	1	0	4

Prey Sleok HC	?	52	120	?	89	264	4	0	0	0	4


*Health center (A): Health center with established PEN, provides screening for CVD, T2D and HTN but treats only HTN. **Health center(B): Health center with established PEN, provides screening for T2D and HTN and treatment for both conditions. ? number of patients were not documented. MW – midwife; MD – medical doctor.

## Costing model

Provider perspective was applied in this costing study; costs to patients, opportunity costs and intangible costs were excluded. We chose this approach to give an idea of government/implementer expenditure in scaling-up the IMIC package. The IMIC costing tool was designed to cost integrated services of CVD prevention, screening, and management at the primary healthcare (health centre) level, and start-up costs including the relevant training of involved healthcare workers [26,27]. For this, we adapted the WHO NCD Costing Tool and added training costs as delineated in the HEART costing tool.

We created the cost modelling using Microsoft Excel, based on service components in Figure 1 and cost elements of the IMIC model. Most input cost data were collected from health facilities that have already implemented the PEN program. Information collected were employee wages and working hours, training expenses, capital expenditures, medical expenses, and other costs (utilities, telephone, internet, fax). Epidemiological data were extracted from existing databases, e.g. STEPS survey 2016 [28]. Where needed input data were unavailable, assumptions were made through extrapolation, for instance, the total number of T2D- and/or HTN-related consultations or contacts with the HC of the catchment population/year (contact rate). The exchange rate 1US$= 4,000 riel was used for currency conversion. The list of input parameters (primary data, secondary data and assumptions) are provided in supplementary file 1 *additional material and tool*.

## Data used for input parameters

### Epidemiological data

The PEN protocol indicates that people aged 40-years or older should undergo routine cardiovascular risk assessment. Therefore, we took the population group aged 40 and above as target population [29]. In as much as each HC reported to cover 8,000–12,000 population, we used the average number (10,000) to estimate the population per catchment area [22]. The proportion of people aged age 40 and above is 27% of the total population [30].

The HC contact rate per year was assumed to be 50% based on the annual number of T2D and/or HTN client visits to the HC, using the estimated population per catchment area as the denominator [31].

The epidemiological estimation for risk distribution was based on data from the Cambodia WHO STEPwise approach to Surveillance (STEPS survey) 2016, where it was noted that 92.30% of adults aged ≥40 years old have at least one risk factor for NCDs [unpublished]. Based on data from literature [cite reference/s], we then assumed that 88.00% of these adults have low CVD risk (0 to <10%), 10% have medium CVD risk (10 to <20%) [28], and 2% have high CVD risk (≥20%) [27]. We based the prevalence of T2D (12%) and HTN (27%) from a recent survey of the SCUBY project in Cambodia [6,7]. Below we provide the epidemiological data and values we used (Table 2).

**Table 2:** Epidemiological data used.


**1. Population Metrics**	**Value**

Population in the area	10,000

% of population age ≥40 years, out of total population	27%

*Population age ≥40 years*,	2700

**2. Coverage rate**	**Value**

Annual health centre contact rate	50%

**3. Epidemiological Data**	

Adults >40 years with at least one risk factor for NCDs	92.30%

Low CVD risk (0 to <10%)	88.00%

Medium CVD risk (10 to <20%)	10.00%

High CVD risk (≥20%)	2.00%

Prevalence of HTN in people aged 40 and above	27.00%

Prevalence of Type 2 Diabetes in adults aged 40 and above	12.00%

Ischemic Heart Disease prevalence	1.72%


### Human resources data

#### Training

Three staff members from each HC participated in a CVD risk screening training to implement PEN [24]; this was applied to the human resource needs for the IMIC. The data used to estimate training costs was provided by the Department of Preventative Medicine of the Ministry of Health (MoH), which is responsible for providing such training sessions. A five-day training includes the following: introduction to PEN, behaviour change and healthy lifestyle, CVD risk assessment, management of T2D and HTN, patients’ records and reporting. Resource people from the MoH served as trainers/lecturers. In addition to training HC staff, community health workers (CHW) in the catchment areas were recruited to join a one-day training for active screening, community campaign/education sessions and follow-up visits/education/counselling. Two trainers from the MoH conducted the training, attended by17 CHWs per HC.

#### Health personnel times and cost

Based on human resource analysis, two full time staff in addition to current staff are needed to perform CVD risk screening, and HTN and T2D management. From verbal interviews and observation during cost data collection regarding turnaround times (TAT), a nurse needs to spend five minutes for triage and registration, while physical examination and consultation (by either medical doctor or nurse) takes 20 minutes per case. TAT for interpretation of laboratory results is 5 minutes. The healthy lifestyle and self-management education session has a TAT of 15 minutes/encounter.

Information on salaries for doctors/nurses were collected to estimate average financial benefits (salary plus all incentives) for each staff. Paid working time was estimated to be 277 days/year, five days/week, eight hours/day. The salary for CHWs was assumed based on the “benefits” provided after decentralization to Village Health Support Group (VHSG) by the Ministry of Interior. CHWs are assumed to work 156 days/year, three days/week and six hours/day.

### Medicine and diagnostic tests data

The types and doses of medicine used in this study followed the PEN SOP for T2D and HTN and Cambodian clinical guidelines for T2D [24,25] confirmed with monthly and annual drug reports collected from health facilities. The numbers of medicines used were extracted from the Central Medical Store (CMS) system. We followed the government’s official price list for medicines, which presumptively included the cost of administration and procurement of the drugs in the final price. While we were allowed access to prices of T2D and HTN drugs for our study, the list is confidential and cannot be made available publicly.

The final price of each laboratory test charged to patients was used to calculate this cost; it was assumed that all associated diagnostic costs were already included.

### Capital costs and other costs data

The capital costs refer to the cost of the building, equipment and vehicles that are used for screening and management of both conditions. The data was collected from the facilities wherein a building was given a 10-year lifespan fixed depreciation. The cost of the building was extracted from the inventory list, and the size of building space needed to deliver the services was measured during data collection. Other costs collected were expenses for utilities, internet, phone, fax as related to the delivery of T2D and HTN care.

## Results

### Time demand on human resources

The estimated number of people aged 40 years or older needing primary care services for T2D and HTN in the population of 10,000 with 50% HC contact rate is 1,246 people. To deliver the IMIC package to the estimated population, medical doctors need to spend 41,653 minutes (87 days), nurses 36,135 minutes (75 days), and CHWs 38,887 minutes (81 days). Therefore, one full time equivalence of each profession including CHW is enough to perform the tasks (Table 3). A doctor spends most of their time on risk screening and physical examination (24,921 minutes) while a nurse spends most of their time on healthy-life style counselling (16,448 minutes). A CHW spends all their time (38,887 minutes) on community/home visits including active screening.

**Table 3:** Time of health personnel in CVD management cases in minutes.


SERVICES	TIME PER CASE PER VISIT (MINUTES)			POPULATION NEEDS SERVICE (PERSON)	TIME SPENT IN A YEAR (MINUTES)		
	
	DOCTOR	NURSE	CHW		DOCTOR	NURSE	CHW

Screening CVD risk with physical exam	20	5	0	1246	24,921	6,230	0

First visit- laboratory screening and risk profiling	10	0	0	1225	12,246	0	0

low risk <10%	0	15	0	1097	0	16,448	0

Medium Risk CVD 10%–20%	10	30	0	125	1,246	3,738	0

High Risk CVD >20%	10	30	0	25	249	748	0

Management of Hypertension	0	0	0	336	0	0	0

management of diabetic patients	20	60	0	150	2,991	8,972	0

Community/home visit	0	0	80	846	0	0	38,877

Annual incremental time (minute)					41,653	36,135	38,887

Annual incremental time (hour)					694	602	648

Annual incremental time (day)					87	75	81


### Cost of the intervention

CVD risk screening costs US$ 1.34/case including costs of physician/nurse time and use of point-of-care glucose test. Laboratory tests for T2D confirmation and CVD risk profiling costs US$ 9.27/case. The annual costs of hypertension and diabetes treatment are US$ 167.90/patient and US$ 69.62/patient respectively. The cost of treating hypertension increases by US$ 2.90 (totalling US$ 170.80/patient) for higher CVD risk (Table 4).

**Table 4:** Annual cost of treatment disaggregated by service in US$.


SERVICE	HUMAN RESOURCE	LABORATORY	COUNSELLING	MEDICINE	CHW	ANNUAL COST PER CASE

Screening CVD risk with physical exam	1.03	0.31	–	–	–	1.34

First visit- laboratory and risk profiling	0.77	8.50	–	–	–	9.27

low risk <10%	–	–	0.62	–	-	0.62

Medium Risk CVD 10%-20%	0.41	1.25	1.24	–	–	2.90

High Risk CVD >20%	0.41	1.25	1.24	–	–	2.90

Management of Hypertension	–	–	–	167.90	–	167.90

Management of diabetic patients	0.82	16.5	2.47	49.82	–	69.62

Follow up visit and counselling by CHWs	–	–	–	–	1.71	1.71


Table 5 shows the annual cost of managing cases in different CVD risk categories. The annual provider-side cost to implement IMIC, including CVD risk screening and profiling, and HTN and T2D management is US$ 82,207.00. The cost of medicine takes the greatest share of about 82% of total costs followed by laboratory tests (13%) and health personnel (5%).

**Table 5:** Annual total cost to treat 10,000 population at 50% health centre contact per year.


SERVICES	UNIT COST US$	NO OF PEOPLE IN NEED OF SERVICE PER 10,000 POP	TOTAL COST PER 10,000 POP US$

Screening CVD risk with physical exam	1.34	1246	1,670.00

First visit- laboratory screening and risk profiling	9.28	1225	11,358.00

low risk <10%	0.62	1097	678.00

Medium Risk CVD 10%–20%	2.90	125	678.00

High Risk CVD >20%	2.90	25	72.00

Management of Hypertension	167.90	336	56,487.00

management of diabetic patients	69.62	150	10,410.00

follow up visit and counselling	1.71	486	831.00

Total IMIC provider-side cost			81,868

IMIC provider-side cost per case (Total direct medical cost/Total case)			66

IMIC provider-side cost per capita (Total direct medical cost/Total population in catchment area)			8

Training cost			752

Material to start up the intervention			1,098

Other cost			805

Total cost			84,523


The other costs including capital and general expenditures is US$ 748.00 and US$ 58.00 respectively and they are assumed to be fixed over time.

The training costs for health personnel and CHWs is US$ 751.00 per health centre with cost per health personnel (doctor, nurse) at US$ 191.00, and US$ 11.00 per CHW.

These costs were calculated per HC with an assumed population of 10,000 in its catchment area. Costs accumulate once with additional 10,000 population (i.e, incremental increase of stated costs for every 10,000 increase in the population).

The total cost of the model is US$ 84,523 per annum including training, equipment to start the services and other costs.

## Discussion

The IMIC model is a costing tool to estimate the cost of integrated care package for T2D and HTN care comprise numbers of key effective interventions including detection, diagnosis, treatment, health education, self-management and follow up. Similar costing tools have been designed and used to project costs of integrated care implementation or scaling up of interventions [32]. However, to our knowledge, cost models for scaling up integrated care packages in resource-constrained settings have not yet been widely explored [33].

The ideal minimum integrated care (IMIC) package costs more than US$ 84,523.00/per HC/annum. Medicine is the main cost driver, consuming approximately 82% of the total annual cost of the intervention. This finding is consistent with a cost study in Rwanda, where medicine was also the major cost driver [34]. Obviously, when the cost of medicine is decreased, the cost of the intervention can be decreased. The high cost of medicine also leads to shortages. To ensure effectiveness of care and adequacy of drug supply, the procurement and drug distribution mechanisms and the final prices of medicines should be reviewed and modified [35].

The estimated cost per eligible case is US$ 66.00; and at US$ 8.00 per capita, based on the assumed number of identified cases utilising health services and the prevalence of T2D (12%) and HTN (27%). However, recruiting more cases into the intervention, which lowers the per capita cost, might affect the quality of care due to increased health personnel workload and, ultimately, inadequate medical materials and supplies.

The current total health expenditure (THE) in Cambodia is US$ 113.00 per capita; adding US$ 8.00 per capita for T2D and HTN increases THE by maximum 7%. Only 23% of the THE is covered by the government; the country’s health financing relies heavily on out-of-pocket expenditure (>60%) [36]. To ensure successful scaling-up of the IMIC and to protect population from financial risk, social health insurance expansion with proper planning and mechanisms in place need to be strongly considered [37].

The cost of services in the IMIC model provides basic cost information to scale-up integrated interventions at national level [34]. With 1,024 health centres [38], scaling-up IMIC in Cambodia will cost >US$86 million. While this surpasses the national spending on NCDs in 2018, it should be noted that IMIC implementation will be on a national scale. The cost of screening including CVD risk screening and glucose rapid testing, which can be conducted by nurses, is low (US$1.34/person screened). A previous study demonstrated that screening is the most cost-effective intervention for early detection averting second-line treatment and complications such as ischemic heart disease for HTN and kidney failure for T2D patients [13]. Additionally, the unit cost for CHWs to serve the assigned service is also low at US$ 1.71/case. CHWs are identified as active agents in many essential health services to improve health status [39]. There is evidence demonstrating that this cadre effectively increase screening for cases, raise awareness of conditions, increase retention in care, and improve HTN and T2D control for those on treatment [40,41]. In a literature review by Vaughan et al., they documented cost-effectiveness of CHWs in several LMICs, for different healthcare interventions including HTN and self-management. Health benefits are thus expected to outweigh the cost, making it worth investing in both the screening strategies and the CHWs. However, such cost-effectiveness studies have not yet been done for Cambodia.

## Strengths and limitations

This work has several limitations. First, the “Ideal Minimum Integrated Care” is a built model inspired by PEN and other effective interventions that attempt to address some identified gaps in current interventions. This model has never been checked against real-world costs. Accuracy of costs projected through this model depends on real-world factors such as human resource capacity, procurement processes and supply of medical materials, and infrastructure. Second, assumptions were made for some parameters such as health centre contact rate, proportion of people with high CVD risk, and personnel leave days, to estimate costs. Third, we assumed that all people would follow the flow of services; in reality, some will be lost to follow-up and drop out in any step of the pathway. Fourth, we assumed that human resource capacity and medicine availability are at the optimum to serve the population. Lastly, this exercise presents with common modelling challenges and is not a full reflection of the real world. Nevertheless, the Excel-based costing tool is easy to accomplish and can be replicated to provide more accurate results by using more precise actual input data, once these are available in the country. The output of the study already provides information on costs of an ideal minimum integrated care package for T2D and HTN to aid decision-making on implementation. Other LMICs with similar contexts and needing information on costs of integrated care especially for evidence-informed decision-making may use or adapt our built-model.

## Lessons learned

- The output of the study provides support towards re-orientation of health systems to implement integrated care to address demands for healthcare amidst resource constraints
- The IMIC cost modelling estimates costs of scaling-up integrated care for HTN and DM in Cambodia and gives decision-makers an idea of investments to prepare in implementing integrated HTN and DM care
- The IMIC model can be utilised to monitor and evaluate actual costs of implementation, once data is available, and can then be compared to national healthcare expenditures
- The IMIC model can be used to project costs of care for any package of health services
- Countries looking into costs of scaling-up (integrated) care packages can also utilise the IMIC model.

## Conclusion

This modelling study gives preliminary evidence on the cost of integrating T2D and HTN detection, diagnosis, treatment + health education, self-management and follow-up at first line health facilities in Cambodia according to our built model “Ideal Minimum Integrated Care”. The cost information will be useful to policy makers for decision-making, planning future health budgets and projecting intervention costs. It may also be useful to international agencies, e.g., the WHO, World Bank, who are looking into financing mechanisms for integrated NCD care in Cambodia. Also, it gives inspiration for the government to revisit other related policies such as health financing and medicine procurement for efficient use of available resources. Our costing tool can also be useful to cost other integrated care interventions beyond T2D and HTN, and to countries with contexts like Cambodia.

## Additional File

The additional file for this article can be found as follows:

10.5334/ijic.7682.s1 Supplementary File 1. Additional material and tool.

## References

[1] World Health Organization. Cardiovascular diseases. Available from: https://www.who.int/health-topics/cardiovascular-diseases#tab=tab_1.

[2] Saeedi P, Petersohn I, Salpea P, Malanda B, Karuranga S, Unwin N, et al. Global and regional diabetes prevalence estimates for 2019 and projections for 2030 and 2045: Results from the International Diabetes Federation Diabetes Atlas. Diabetes research and clinical practice. 2019; 157: 107843. DOI: 10.1016/j.diabres.2019.107843 31518657

[3] Zhou B, Carrillo-Larco RM, Danaei G, Riley LM, Paciorek CJ, Stevens GA, et al. Worldwide trends in hypertension prevalence and progress in treatment and control from 1990 to 2019: a pooled analysis of 1201 population-representative studies with 104 million participants. The Lancet. 2021; 398(10304): 957–80. DOI: 10.1016/S0140-6736(21)01330-1 PMC8446938 34450083

[4] World Health Organization. Diabetes. Available from: https://www.who.int/news-room/fact-sheets/detail/diabetes.

[5] World Health Organization. Hypertension Available from: https://www.who.int/news-room/fact-sheets/detail/hypertension.

[6] Chhim S, Te V, Buffel V, Van Olmen J, Chham S, Long S, et al. Healthcare utilization and expenditure among people with type 2 diabetes and/or hypertension in Cambodia: results from a cross-sectional survey. BMJ Open. 2023. DOI: 10.1136/bmjopen-2022-061959 PMC9843177 36635032

[7] Chham S, Buffel V, Van Olmen J, Chhim S, Ir P, Wouters E. The cascade of hypertension care in Cambodia: evidence from a cross-sectional population-based survey. BMC Health Services Research. 2022; 22(1): 838. DOI: 10.1186/s12913-022-08232-7 35768805 PMC9241312

[8] Seuring T, Archangelidi O, Suhrcke M. The economic costs of type 2 diabetes: a global systematic review. Pharmacoeconomics. 2015; 33: 811–31. DOI: 10.1007/s40273-015-0268-9 25787932 PMC4519633

[9] Sorato MM, Davari M, Kebriaeezadeh A, Sarrafzadegan N, Shibru T. Societal economic burden of hypertension at selected hospitals in southern Ethiopia: a patient-level analysis. BMJ open. 2022; 12(4): e056627. DOI: 10.1007/s40273-015-0268-9 PMC8987749 35387822

[10] World Health Organization. Prevention and control of noncommunicable diseases in Cambodia The case for investment; 2019.

[11] Fuchs FD, Whelton PK. High blood pressure and cardiovascular disease. Hypertension. 2020; 75(2): 285–92. DOI: 10.1161/HYPERTENSIONAHA.119.14240 31865786 PMC10243231

[12] van Olmen J, Menon S, Poplas Susič A, Ir P, Klipstein-Grobusch K, Wouters E, et al. Scale-up integrated care for diabetes and hypertension in Cambodia, Slovenia and Belgium (SCUBY): a study design for a quasi-experimental multiple case study. Global Health Action. 2020; 13(1): 1824382. DOI: 10.1080/16549716.2020.1824382 33373278 PMC7594757

[13] Herman WH, Ye W, Griffin SJ, Simmons RK, Davies MJ, Khunti K, et al. Early detection and treatment of type 2 diabetes reduce cardiovascular morbidity and mortality: a simulation of the results of the Anglo-Danish-Dutch study of intensive treatment in people with screen-detected diabetes in primary care (ADDITION-Europe). Diabetes care. 2015; 38(8): 1449–55. DOI: 10.2337/dc14-2459 25986661 PMC4512138

[14] World Health Organization. Implementation of a package of essential noncommunicable (PEN) disease interventions in Kyrgyzstan: evaluation of effects and costs in Bishkek after one year; 2017.

[15] National Institute of Public Health. Policy brief: Scaling-up integrated care package for type 2 diabetes and hypertension in cambodia: Evidence and policy consideration. Phnom Penh: National Institute of Public Health 2021; 2021.

[16] Tripathy JP, Mishra S. How effective was implementation of the package of essential non-communicable disease (PEN) interventions: A review of evidence? Diabetes & Metabolic Syndrome: Clinical Research & Reviews. 2021; 15(5): 102266. DOI: 10.1016/j.dsx.2021.102266 34496339

[17] Sharma M, John R, Afrin S, Zhang X, Wang T, Tian M, et al. Cost-effectiveness of population screening programs for cardiovascular diseases and diabetes in low-and middle-income countries: a systematic review. Frontiers in public health. 2022; 10: 820750. DOI: 10.3389/fpubh.2022.820750 35345509 PMC8957212

[18] Howard K, White S, Salkeld G, McDonald S, Craig JC, Chadban S, et al. Cost-Effectiveness of screening and optimal management for diabetes, hypertension, and chronic kidney disease: a modeled analysis. Value in health. 2010; 13(2): 196–208. DOI: 10.1111/j.1524-4733.2009.00668.x 19878493

[19] Nazar CMJ, Bojerenu MM, Safdar M, Marwat J. Effectiveness of diabetes education and awareness of diabetes mellitus in combating diabetes in the United Kigdom; a literature review. Journal of Nephropharmacology. 2016; 5(2): 110. 28197516 PMC5297564

[20] Mishra SR, Neupane D, Preen D, Kallestrup P, Perry HB. Mitigation of non-communicable diseases in developing countries with community health workers. Globalization and health. 2015; 11: 1–5. DOI: 10.1186/s12992-015-0129-5 26555199 PMC4641398

[21] Flanagan S, Damery S, Combes G. The effectiveness of integrated care interventions in improving patient quality of life (QoL) for patients with chronic conditions. An overview of the systematic review evidence. Health and quality of life outcomes. 2017; 15: 1–11. DOI: 10.1186/s12955-017-0765-y 28962570 PMC5622519

[22] Ministry of Health. Health Strategic Plan Phnom Penh, Cambodia Ministry of health 2016–2020.

[23] Jacobs B, Hui K, Lo V, Thiede M, Appelt B, Flessa S. Costing for universal health coverage: insight into essential economic data from three provinces in Cambodia. Health Economics Review. 2019; 9: 1–14. DOI: 10.1186/s13561-019-0246-6 31667671 PMC6822335

[24] Ministry of Health. National standard operating procedure for diabetes and hypertension management in primary care 2019. Phnom Penh, Cambodia Ministry of Health; 2019.

[25] Ministry of Health. Clinical Practice Guidelines For Type 2 Diabetes. Phnom Penh, Cambodia Ministry of Health; 2015.

[26] World Health Organization. From burden to “Best Buys”: reducing the economic impact of non-communicable diseases; 2011.

[27] TEPHINET. The HEARTS Costing Tool User Manual Version 1.0. Atlanta, Georgia, USA; 2021.

[28] University of Health Science: National Non Communicable Diseases Risk Factor Survey 2016. Phnom Penh, Cambodia University of Health Science; 2016.

[29] Lim SS, Gaziano TA, Gakidou E, Reddy KS, Farzadfar F, Lozano R, et al. Prevention of cardiovascular disease in high-risk individuals in low-income and middle-income countries: health effects and costs. The Lancet. 2007; 370(9604): 2054–62. DOI: 10.1016/S0140-6736(07)61699-7 18063025

[30] Ministry of Planning: General Population Census of the Kingdom of Cambodia 2019. Phnom Penh Cambodia National Institution of Statistics; 2019.

[31] Ministry of Health. Cambodia Health Management Information System. Phnom Penh, Cambodia; 2022.

[32] d’Elbée M, Terris-Prestholt F, Briggs A, Griffiths UK, Larmarange J, Medley GF, et al. Estimating health care costs at scale in low-and middle-income countries: Mathematical notations and frameworks for the application of cost functions. Health economics. 2023; 32(10): 2216–33. DOI: 10.1002/hec.4722 37332114

[33] Collins D, Griffiths U, Birse S, Dukhan Y, Bocoum FY, Driwale A, et al. Calculating the Costs of Implementing Integrated Packages of Community Health Services: Methods, Experiences, and Results From 6 sub-Saharan African Countries. Global Health: Science and Practice. 2023; 11(5). DOI: 10.9745/GHSP-D-22-00472 PMC10615248 37903585

[34] Eberly LA, Rusangwa C, Neal CC, Mukundiyukuri JP, Mpanusingo E, Mungunga JC, et al. Cost of integrated chronic care for severe non-communicable diseases at district hospitals in rural Rwanda. BMJ Global Health. 2019; 4(3): e001449. DOI: 10.1136/bmjgh-2019-001449 PMC6597643 31321086

[35] Nang EEK, Dary C, Hsu LY, Sor S, Saphonn V, Evdokimov K. Patients’ and healthcare providers’ perspectives of diabetes management in Cambodia: a qualitative study. BMJ open. 2019; 9(11): e032578. DOI: 10.1136/bmjopen-2019-032578 PMC6887069 31753894

[36] World Health Organization. Cambodia national health accounts (2012–2016): health expenditure report. 2019; Report No. 9290618698.

[37] Kolesar RJ, Pheakdey S, Jacobs B, Chan N, Yok S, Audibert M. Expanding social health protection in Cambodia: An assessment of the current coverage potential and gaps, and social equity considerations. International Social Security Review. 2020; 73(1): 35–63. DOI: 10.1111/issr.12227

[38] World Health Organization. The Kingdom of Cambodia health system review. 2015. Report No. 9290616911.

[39] Vaughan K, Kok MC, Witter S, Dieleman M. Costs and cost-effectiveness of community health workers: evidence from a literature review. Human resources for health. 2015; 13: 1–16. DOI: 10.1186/s12960-015-0070-y 26329455 PMC4557864

[40] McGuire H, Van TB, Thi Thu HL, Nguyen Thanh H, Murray M, Shellaby J, et al. Improving hypertension awareness and management in Vietnam through a community-based model. Scientific Reports. 2022; 12(1): 19860. DOI: 10.1038/s41598-022-22546-w 36400798 PMC9673873

[41] Nikpour Hernandez N, Ismail S, Heang H, van Pelt M, Witham MD, Davies JI. An innovative model for management of cardiovascular disease risk factors in the low resource setting of Cambodia. Health Policy and Planning. 2021; 36(4): 397–406. DOI: 10.1093/heapol/czaa176 33367513 PMC8128014

